# Polymorphisms of Renin-Angiotensin-Aldosterone System Gene in Chinese Han Patients with Nonfamilial Atrial Fibrillation

**DOI:** 10.1371/journal.pone.0117489

**Published:** 2015-02-27

**Authors:** Li-qun Zhao, Zu-jia Wen, Yong Wei, Juan Xu, Zheng Chen, Bao-zhen Qi, Zhi-ming Wang, Yong-yong Shi, Shao-wen Liu

**Affiliations:** 1 Department of Cardiology, Shanghai First People’s Hospital Affiliated to Shanghai JiaoTong University, Shanghai 200080, China; 2 Bio-X Institutes, Shanghai Jiao Tong University, Shanghai, 200030, China; 3 Department of Genetics, Shanghai-MOST Key Laboratory of Health and Disease Genomics, Chinese National Human Genome Center and Shanghai Industrial Technology Institute (SITI), Shanghai 201203, China; Max-Delbrück Center for Molecular Medicine (MDC), GERMANY

## Abstract

**Background:**

Atrial fibrillation(AF) is the most common arrhythmia in the adult population. The activated renin-angiotensin-aldosterone system (RAS) has been reported to play an important role in the pathogenesis of atrial fibrillation. The aim of this study was to investigate the association between nonfamilial AF and polymorphisms in RAS gene.

**Methods:**

A total of 931 patients with nonfamilial AF, 663 non-AF heart disease patients and 727 healthy subjects were selected. 10 tagSNPs (tSNPs) (ACE gene rs8066114, AGT gene rs7539020, rs3789678, rs2478544, rs11568023, rs2478523, rs4762, rs699 and CYP11B2 rs3802230, rs3097) were chosen and genotyped in our study. Single-locus analysis and haplotype analysis were used in this study.

**Results:**

In single-locus analysis, we found rs11568023 and rs3789678 in AGT gene were associated with nonfamilial AF in Chinese Han population. AF risk was associated with rs3789678 between the AF group and control groups. Under dominant model, the significant AF risk was observed in rs3789678 between the AF group and non AF heart control group; And the protective effect was found in rs11568023, compared with the non-AF heart disease control group. In multilocus haplotype analysis, the association between frequencies of the haplotypes and AF risk was showed in AGT gene (rs7539020-rs3789678), compared ‘TT’ haplotype with the common ‘TC’ haplotype, adjusted for age, gender, LVEF, LVEDD, LAD and frequency of hypertension and diabetes. The diplotype with ‘TC’, carrying rs3789678-C-allele, was associated with reduced risk of AF between the AF group and the healthy control group. The diplotype with ‘TT’ haplotype in the same block, carrying rs3789678-T-allele, was associated with increased risk of AF.

**Conclusions:**

Via a large-scale case-control study, we found that rs3789678 site was potential susceptible locus of AF whereas rs11568023 was protective factor.

## Introduction

Atrial fibrillation is the most common arrhythmia in the adult population. However, the underlying mechanisms of AF remain unknown. So far a lot of evidence is in support of a role for renin-angiotensin-aldosterone system (RAS) activation as an important factor in paroxysmal and persistent AF. Genetic variations of RAS affect plasma and tissue RAS activity, and ACE and angiotensin II levels. Hiroshi Watanabe [1] demonstrated for the first time that the ACE I/D polymorphism is associated with atrial and atrioventricular conduction in patients with lone AF. Polymorphisms in the angiotensinogen gene were also associated with AF as they might cause higher angiotensinogen gene transcription activity and a higher tissue angiotensin II concentration in the atrium under the stimulation of high atrial pressure [2, 3]. However, Yamashita et al. [4] examined an Asian cohort with lone AF and found no significant association between the DD genotype and AF. The results of a meta-analysis indicated that there was insufficient evidence to draw clear conclusions on the potential association between the ACE I/D polymorphism and AF risk [5].

Some studies suggest that polymorphisms in the renin-angiotensin- aldosterone system alter activation of the renin-angiotensin- aldosterone system and increase the likelihood of atrial fibrillation. However, they are far from being definitive. The majority of studies about commonly occurring SNPs have not been replicated in independent populations or in different ethnic groups and have been substantially underpowered. Case-control studies are useful in identifying gene polymorphisms that are associated with disease. If the polymorphism itself is functional, or an allele polymorphism is in linkage disequilibrium with a disease susceptibility allele, we will find functional mutation and discover the mechanism of atrial fibrillation. Based upon the aforementioned studies on RAS genes and AF, we hypothesized that RAS genes may be the susceptible genes of non-familial AF, and we can find the association between the RAS gene variantsandnon-familial AF in a matched case-control study. Therefore, we investigated the association of tSNPs in RAS gene (ACE gene rs8066114, AGT gene rs7539020, rs3789678, rs2478544, rs11568023, rs2478523, rs4762, rs699 and CYP11B2 rs3802230, rs3097) and atrial fibrillation in a Chinese Han sample.

## Materials and Methods

### Study population

This study was approved by the Institutional Review Board of Shanghai First People’s Hospital affiliated to Shanghai Jiao Tong University. All patients provided written informed consent. We enrolled 931AF subjects as AF group (529 men and 402 women; mean age, 64.63± 10.83 years) and 663 non-AF heart disease subjects as control group 1 (363 men and 300 women; mean age, 64.96±7.90 years), admitted to department of Cardiology from July 2012 to August 2013. We also enrolled 727 healthy subjects as control group 2 (378 men and 349 women; mean age, 61.81±8.38 years) from our medical examination center in 2012 (Table 1, 2). All of three groups came from Shanghai First People’s Hospital affiliated to Shanghai Jiao Tong University. AF was diagnosed by electrocardiogram (ECG) or Holter. Exclusion criteria for case group included one of the following: familial AF, recent MI (six months or less), cardiac surgery (30 days or less), New York Heart Association class III or IV, severe valve disease, thyroid dysfunction, renal or lung dysfunction, and AF due to trauma, surgery, or acute medical illness. For every case patient, a matched control group1 without a history of AF was selected from the same ward. Case and control group1 patients were individually matched with regard to their gender, age (difference less than 5 years), New York Heart Association class, valvular heart disease, acute medical illness, hypertension and diabetes. All subjects in the case group and control groups were less than 80 years old. We elected healthy control group without AF history from our hospital’s medical examination center. Their electrocardiogram (ECG) and Holter showed sinus rhythm.

**Table 1:** Baseline clinical characteristics of AF group and non-AFheart disease control group.

	AF patient (931)	Control 1 (663)	P
Sex(M/F)	529/402	363/300	0.542
Age(years)	64.63±10.83	64.96±7.90	0.912
LAD, cm	42.93±4.97	40.34±6.87	**0.000**
LVEDD, cm	50.25±4.31	48.38±4.73	**0.000**
LVEF %	59.85±6.43	60.51±5.58	**0.014**
Hypertenion	36.8%	39.6%	0.225
Diabetes	7.70%	8.30%	0.656

LAD-left atrial dimension

LVEDD-left ventricular end diastolic dimension

LVEF-left ventricular ejection fraction

Control 1-non-AFheart disease group

**Table 2:** Baseline clinical characteristics of AF group and healthy control group.

	AF patient (931)	Control 2 (727)	P
Sex(M/F)	529/402	378/349	0.050
Age(years)	64.63±10.83	61.81±8.38	**0.000**
LAD, cm	42.93±4.97	35.49±4.69	**0.000**
LVEDD, cm	50.25±4.31	48.36±4.50	**0.000**
LVEF %	59.85±6.43	61.85±5.78	**0.000**
Hypertension	36.8%	-	
Diabetes	7.70%	-	

LAD-left atrial dimension

LVEDD-left ventricular end diastolic dimension

LVEF- left ventricular ejection fraction

Control 2-healthy group

Transthoracic echocardiography was performed to measure the left atrial and left ventricular dimensions and to detect significant valvular disease (defined as moderate to severe valvular regurgitation or stenosis, normal left atrial dimension 19–40 mm, normal left ventricular end-systolic dimension 20–40 mm, left ventricular end-diastolic dimension 35–55 mm).

Three groups were comparable with regard to ethnicity, sex, and residence area. However, the factors of age, left atrium dimension, left ventricular end diastolic dimension and left ventricular ejection fraction showed significant differences between the AF group and control groups (Table 1, 2)

### Extraction of genomic DNA

A 2-mL volume of venous blood samples from each participant was taken in citrate-anticoagulated glass tubes, and was frozen at -40°C. Total genomicDNA of the leucocyte was extracted from 1 ml of peripheral blood using the Whole Blood DNA Extraction Kit (QIAamp DNA Blood Mini Kit), according to the manufacturer’s instructions. Genomic DNA extracted was dissolved in 0.1×TE buffer (10 mMTris–1 mM EDTA, pH8.0) and stored at -20°C.

### TagSNP selection

Some tSNPs of RAS system gene were selected by using genotype data obtained from the International HapMap Project (http://hapmap.ncbi.nlm.nih.gov) data (released # 27/PhaseII+III Feb 09). To identify common tagging SNPs, these eligible SNPs were entered into the Tagger program implemented in Haploview 4.2 program (http://www.broad.mit.edu/haploview/haploview-downloads). We defined the common variants as those with minor allele frequency (MAF) of more than 5% and set the threshold of 0.8 for LD measure r^2^. In the end, a total of 10 tSNPs (ACE gene rs8066114, AGT gene rs7539020, rs3789678, rs2478544, rs11568023, rs2478523, rs4762, rs699 and CYP11B2 rs3802230, rs3097) were chosen in our study.

### SNP genotyping assays

SNPs were typed using iPLEX chemistry on a matrix-assisted laser desorption/ionization time-of-flight mass spectrometer (MALDI-TOF-MS, named as MassARRAY system, manufactured by Sequenom, Inc.). In brief, (1) multiplex PCR amplification: PCR reactions were carried out in standard 384-well plates in 5 μL per reaction with 10 ng of genomic DNA, 0.5 units of Taq polymerase (HotStarTaq, Qiagen), 500 μmol of each deoxynucleotidetriphosphate (dNTP), and 100 nmol of each PCR primer. PCR thermal cycling was carried out in an ABI-9700 instrument for 15 min at 94°C, followed by 45 cycles of 20s at 94°C, 30s at 56°C, and 60s at 72°C. PCR products were electrophoresed on 2.0% agarose. (2) Removing residual primers and dNTPs: after PCR reaction, 2 μL containing 0.3 units of Shrimp Alkaline Phosphatase was added, and the reaction was incubated at 37°C for 20 min followed by inactivation for 5 min at 85°C. (3) PCR with single base extension: After adjusting the concentrations of extension primers to equilibrate signal-to-noise ratios, the post-PCR primer extension reaction of the iPLEX Gold Kits (Sequenom, Inc.) assay was done in a final 9 μL volume extension reaction containing 0.2 μL (100 μmol) of termination mix, 0.04 μL containing 0.05 units of DNA polymerase (Sequenom, Inc.), and 625 to 1,250 nmol/L extension primers. A 200-short-cycle program was used for the iPLEX reaction: initial denaturation was for 30s at 94°C followed by 5s at 94°C and five cycles of 5s at 52°C and 5s at 80°C. An additional 40 annealing and extension cycles were then looped back to 5s at 94°C, five cycles of 5s at 52°C and 5s at 80°C. The final extension was carried out at 72°C for 3 min and the sample was cooled to 4°C. (4) Analyses of purified extension reaction products by MALDI-TOF-MS: The samples were then manually desalted by using 6 mg of clean resin and a dimple plate and subsequently transferred to a 384-well Spectro-CHIP (Sequenom, Inc.) using a nanodispenser. Mass spectrum was acquired by Compact Mass Spectrometer and analyzed by MassARRAYTyper 4.0 Software (Sequenom, Inc.). The PCR assay was arrayed with two no-template controls and four duplicated samples in each 384-well format as quality controls. All genotyping results were generated and checked by laboratory staff unaware of patient status.

### Statistical analyses

The statistical analyses were performed with the Stata statistical package (version 10.0; StataCorp LP, College Station, TX, USA). The quantitative variables were presented as the mean ± SD, which are normally distributed. Measured variables were compared in patients with and without AF and the healthy group by an unpaired Student’s t-test. Hardy-Weinberg Equilibrium (HWE) was tested in the healthy group by the chi-square test. The allele frequency, genotype, haplotype as well as dihaplotype between the groups were examined by unconditional logistic regression analysis with adjustment for age, gender, LVEF, LVEDD, LAD and frequency of hypertension and diabetes. P < 0.05 was considered statistically significant.

The pairwise linkage disequilibrium (LD) among the SNPs was examined using Lewontin’s standardized coefficient D’ and LD coefficient r^2^ [6], and haplotype blocks were defined by the method of Gabriel et al. [7] in Haploview 4.2 with default settings (the CI for a strong LD was minimal for upper 0.98 and low 0.7 and maximal for a strong recombination of 0.9, and a fraction of strong LD in informative comparisons was at least 0.95). In addition, PHASE 2.1 Bayesian algorithm [8] was used to estimate the haplotype frequencies. The HAPLO.STATS package developed by Schaidet al. [9] (http://www.mayo.edu/hsr/Sfunc.html) in the software language R was used for the haplotype analysis. Haplotypes with a frequency of less than 0.03 were pooled into a combined group. Diplotype (haplotype dosage, an estimate of the number of copies of the haplotype) was the most probable haplotype pair for each individual. Unconditional logistic regression analyses were conducted to estimate ORs and 95% CIs for participants carrying one to two copies versus zero copy of each common haplotype for the dichotomized diplotypes.

## Results

### Individual SNP association analysis

There was no deviation from the Hardy-Weinberg equilibrium in the healthy group. After Bonferroni correction (the results were corrected for SNP number and the number of statistical analyses), the allele frequencies of AGT gene SNP5 rs11568023 was significantly different between the AF group and non-AF heart disease control group (P = 0.0003). And the allele frequencies of AGT gene SNP7 rs3789678 was significantly different between the AF group and the healthy control group (P = 0.043) (Table 3).

**Table 3:** Information of 10 genotyped SNPs of AGT,CYP11B2 and ACEgenes.

Gene: locus and OMIM No.^a^	No.	SNP_ID	Chromosome No.	Chromosome Position ^b^	Intermarker distances (bp)	Genic location	Base Change	MAF ^c^			P ^f^	P^g^ value for HWE test	Genotyping Rate (%)
								NCBI ^d^	control^e^	AF			
AGT: 1q42.2 OMIN:106150	1	rs2478523	1	230841509	2687	intron	C/T	0.407	0.436/0.457	0.470	0.627/1.000	0.6148/1.000	95.00
	2	rs2478544	1	230844196	1598	intron	G/C	0.222	0.226/0.244	0.230	1.000/1.000	0.6877/1.000	95.35
	3	rs699	1	230845794	183	Exon 2 M268T	C/T	0.279	0.171/0.185	0.172	1.000/1.000	1.000/1.000	84.36
	4	rs4762	1	230845977	2202	Exon 2 T207M	C/T	0.081	0.110/0.090	0.104	1.000/1.000	0.1029/1.000	95.00
	5	rs11568023	1	230848179	1011	intron	C/T	0.057	0.109/0.071	0.067	**0.0003**/1.000	0.1396/1.000	98.06
	6	rs7539020	1	230849190	292	intron	T/C	0.400	0.323/0.310	0.229	1.000/1.000	0.5677/1.000	96.21
	7	rs3789678	1	230849482	——	intron	C/T	0.156	0.160/0.161	0.200	0.050/**0.043**	**0.0345/**0.345	96.12
CYP11B2: 8q24.3OMIN: 124080	1	rs3802230	8	143992864	451	3’ UTR	C/A	0.349	0.267/0.300	0.267	1.000/0.351	0.5907/1.000	99.05
	2	rs3097	8	143993315	——	3’ UTR	G/A	0.056	0.092/0.058	0.073	0.653/0.855	0.2916/1.000	97.24
ACE: 17q23.3OMIN: 612624	1	rs8066114	17	61589840	——	3’ near gene	C/G	0.186	0.215/0.236	0.198	1.000/0.102	0.4339/1.000	95.13

a. OMIM, Online Mendelian Inheritance in Man (http://www.ncbi.nlm.nih.gov/Omim).

b. SNP position in the NCBI dbSNP Build 37 database (http://www.ncbi.nlm.nih.gov/SNP).

c. MAF, minor allele frequency

d. MAF for Chinese in the NCBI dbSNPs database.

e. MAF for non-AF heart disease control/healthy control group

f. P value for difference in allele distributions between AFand non-AF heart disease control/healthy control group after Bonferroni correction.

g. P value for HWE, Hardy–Weinberg equilibrium in non-AF heart disease control/healthy controlgroupbefore and after Bonferroni correction.

After Bonferroni correction, unconditional logistic regression analyses revealed that AF risk was associated with AGT SNP7 rs3789678, compared with the non-AF heart disease control group (P = 1.28E-03, OR = 1.678, 95% CI = 1.313–2.146 for CT/CC), adjusted for age, gender, LVEF, LVEDD, LAD and frequency of hypertension and diabetes (Table 4).

**Table 4:** Genotype distribution of 10 genotyped SNPs of AGT, CYP11B2andACE genes between AF group and non-AF heart disease control/healthy control group.

Gene	SNP no.	SNP ID	Genotype	Case		Control1^d^		Control 2^e^		Logistic Regression^a^		Logistic Regression^b^	
				No.	Frequency	No.	Frequency	No.	Frequency	OR (95%CI)	P^c^	OR (95%CI)	P^c^
AGT	1	rs2478523	TT	276	31.58%	217	34.78%	205	29.04%	1.000 (referent)		1.000 (referent)	
			TC	374	42.79%	270	43.27%	357	50.57%	1.099 (0.859–1.406)	0.453/1.000	0.801 (0.607–1.056)	0.115/1,000
			CC	224	25.63%	137	21.96%	144	20.40%	1.286 (0.964–1.716)	0.087/1.000	1.158 (0.837–1.602)	0.377/1.000
	2	rs2478544	GG	542	61.04%	376	60.94%	407	57.49%	1.000 (referent)		1.000 (referent)	
			GC	283	31.87%	203	32.90%	257	36.30%	0.940 (0.745–1.186)	0.603/1.000	0.849 (0.661–1.092)	0.203/1.000
			CC	63	7.09%	38	6.16%	44	6.21%	1.072 (0.692–1.658)	0.756/1.000	0.909 (0.566–1.460)	0.694/1.000
	3	rs699	CC	534	69.62%	339	66.73%	470	68.81%	1.000 (referent)		1.000 (referent)	
			CT	202	26.34%	150	29.53%	193	28.26%	0.850 (0.654–1.104)	0.224/1.000	0.867 (0.660–1.140)	0.306/1.000
			TT	31	4.04%	19	3.74%	20	2.93%	0.989 (0.533–1.835)	0.971/1.000	1.302 (0.639–2.654)	0.467/1.000
	4	rs4762	CC	713	80.93%	507	79.84%	572	83.38%	1.000 (referent)		1.000 (referent)	
			CT	153	17.37%	116	18.27%	105	15.31%	0.871 (0.660–1.149)	0.327/1.000	1.028 (0.745–1.419)	0.864/1.000
			TT	15	1.70%	12	1.89%	9	1.31%	1.035 (0.465–2.304)	0.933/1.000	1.523 (0.559–4.149)	0.410/1.000
	5	rs11568023	CC	799	88.19%	531	81.82%	620	85.99%	1.000 (referent)		1.000 (referent)	
			CT	92	10.15%	94	14.48%	100	13.87%	0.666 (0.484–0.916)	**0.012**/0.360	0.709 (0.500–1.006)	0.054/1.000
			TT	15	1.66%	24	3.70%	1	0.14%	0.467 (0.237–0.921)	**0.028/**0.560	12.865 (1.579–104.790)	**0.017**/0.340
	6	rs7539020	TT	452	50.56%	293	47.41%	340	47.16%	1.000 (referent)		1.000 (referent)	
			TC	350	39.15%	251	40.61%	315	43.69%	0.912 (0.726–1.145)	0.427/1.000	0.854 (0.667–1.094)	0.211/1.000
			CC	92	10.29%	74	11.97%	66	9.15%	0.774 (0.544–1.101)	0.154/1.000	1.011 (0.672–1.522)	0.958/1.000
	7	rs3789678	CC	568	63.61%	447	72.45%	500	69.35%	1.000 (referent)		1.000 (referent)	
			CT	293	32.81%	143	23.18%	210	29.13%	**1.678 (1.313–2.146)**	**6.42E-05/1.28E-03**	1.335 (1.034–1.725)	**0.027**/0.540
			TT	32	3.58%	27	4.38%	11	1.53%	1.033 (0.599–1.782)	0.906/1.000	2.368 (1.127–4.976)	**0.023**/0.460
CYP11B2	1	rs3802230	CC	509	54.91%	344	51.89%	350	49.37%	1.000 (referent)		1.000 (referent)	
			CA	341	36.79%	284	42.84%	292	41.18%	0.807 (0.649–1.003)	0.054/1.000	0.699 (0.546–0.895)	**0.005**/0.100
			AA	77	8.31%	35	5.28%	67	9.45%	1.577 (1.013–2.455)	**0.044**/1.000	0.833 (0.545–1.274)	0.399/1.000
	2	rs3097	GG	774	86.10%	539	84.62%	641	88.90%	1.000 (referent)		1.000 (referent)	
			GA	118	13.13%	79	12.40%	76	10.54%	1.036 (0.755–1.421)	0.829/1.000	1.136 (0.795–1.624)	0.482/1.000
			AA	7	0.78%	19	2.98%	4	0.55%	0.326 (0.133–0.795)	**0.014**/0.280	2.539 (0.551–11.705)	0.232/1.000
ACE	1	rs8066114	CC	569	65.03%	380	62.30%	426	58.92%	1.000 (referent)		1.000 (referent)	
			CG	265	30.29%	198	32.46%	253	34.99%	0.907 (0.717–1.146)	0.412/1.000	0.808 (0.628–1.041)	0.099/1.000
			GG	41	4.69%	32	5.25%	44	6.09%	0.856 (0.522–1.403)	0.537/1.000	0.803 (0.474–1.360)	0.415/1.000

a. Analyses based on non-AF heart disease control group

b. Analyses based on healthy control group

c. P values from unconditional logistic regression analyses, adjusted for age, gender LVEF, LVEDD,LAD and frequency of hypertension and diabetes before and after Bonferroni correction.

d. control1-non-AF heart disease control group

e. control2-healthy control group

Under dominant model, the significant AF risk was observed in AGT SNP7 rs3789678, compared with the non-AF heart disease control group (P = 6.40E-03, OR = 1.573, 95% CI = 1.246–1.986 for CT+TT vs.CC). And the protective effect was found in SNP5 rs11568023 compared with the non-AF heart disease control group (P = 0.040, OR = 0.627, 95% CI = 0.467–0.841for TC+TT vs.CC) (Table 5).

**Table 5:** Association analysis of 10 genotyped SNPs of AGT, CYP11B2 and ACE gene under dominant and recessive genetic model.

Gene	SNP no.	SNP ID	Genetic model	Case	Control 1	Control 2	Logistic Regression		Logistic Regression	
							OR (95%CI)	P^a^	OR (95%CI)	P^b^
AGT	1	rs2478523	CT+CC vs.TT	598/276	407/217	501/205	1.164 (0.928–1.460)	0.189/1.000	0.909 (0.702–1.175)	0.465/1.000
			CC vs.TT+CT	224/650	137/487	144/562	1.218 (0.946–1.567)	0.126/1.000	1.324 (1.002–1.748)	**0.048**/0.960
	2	rs2478544	GC+CC vs.GG	346/542	241/376	301/407	0.964 (0.774–1.201)	0.747/1.000	0.859 (0.677–1.090)	0.211/1.000
			CC vs.GG+GC	63/825	38/579	44/664	1.094 (0.712–1.680)	0.681/1.000	0.965 (0.607–1.536)	0.881/1.000
	3	rs699	CT+TT vs.CC	233/534	169/339	213/470	0.865 (0.674–1.112)	0.258/1.000	0.903 (0.694–1.175)	0.449/1.000
			TT vs.CC+CT	31/736	19/489	20/663	1.037 (0.562–1.914)	0.908/1.000	0.903 (0.694–1.175)	0.449/1.000
	4	rs4762	TC+TT vs.CC	168/713	128/507	114/572	0.883 (0.677–1.153)	0.362/1,000	1.063 (0.779–1.450)	0.702/1.000
			TT vs.CC+TC	15/866	12/623	9/677	1.059 (0.477–2.353)	0.888/1,000	1.516 (0.557–4.124)	0.415/1.000
	5	rs11568023	TC+TT vs.CC	107/799	118/531	101/620	0.627 (0.467–0.841)	**0.002/0.040**	0.829 (0.592–1.162)	0.276/1.000
			TT vs.CC+TC	15/891	24/625	1/720	0.491 (0.249–0.967)	**0.040**/0.800	13.431 (1.650–109.333)	**0.015**/0.300
	6	rs7539020	CT+CC vs.TT	442/452	325/293	381/340	0.881 (0.712–1.091)	0.247/1.000	0.882 (0.698–1.115)	0.294/1.000
			CC vs.TT+CT	92/802	74/544	66/655	0.814 (0.581–1.142)	0.234/1.000	1.088 (0.735–1.611)	0.674/1.000
	7	rs3789678	CT+TT vs.CC	325/568	170/447	221/500	1.573 (1.246–1.986)	**3.20E-04/6.40E-03**	1.402 (1.094–1.798)	**0.008**/0.160
			TT vs.CC+CT	32/861	27/590	11/710	0.888 (0.517–1.524)	0.666/1.000	2.161 (1.033–4.522)	**0.041**/0.820
CYP11B2	1	rs3802230	CA+AA vs.CC	418/509	319/344	359/350	0.891 (0.723–1.097)	0.276/1.000	0.722 (0.572–0.912)	**0.006**/0.120
			AA vs.CC+CA	77/850	35/628	67/642	1.725 (1.119–2.658)	**0.013**/0.260	0.968 (0.642–1.461)	0.878/1.000
	2	rs3097	AG+AAvs.GG	125/774	98/539	80/641	0.908 (0.675–1.223)	0.527/1.000	1.183 (0.834–1.677)	0.346/1.000
			AAvs.GG+AG	7/892	19/618	4/717	0.324 (0.133–0.790)	**0.013**/0.260	2.503 (0.543–11.538)	0.239/1.000
ACE	1	rs8066114	CG+GG vs.CC	306/569	230/380	297/426	0.898 (0.718–1.122)	0.342/1.000	0.807 (0.635–1.027)	0.082/1.000
			GG vs.CC+CG	41/834	32/578	44/679	0.882 (0.541–1.438)	0.616/1.000	0.864 (0.514–1.452)	0.580/1.000

a. P values from unconditional logistic regression analyses between AF group and non-AF heart disease control group (control 1), adjusted for age, gender LVEF, LVEDD, LAD and frequency of hypertension and diabetes before and after Bonferroni correction.

b. P values from unconditional logistic regression analyses between AF group and healthy control group (control 2).

### LD Analysis and Haplotype Block Structure

**Fig 1:**
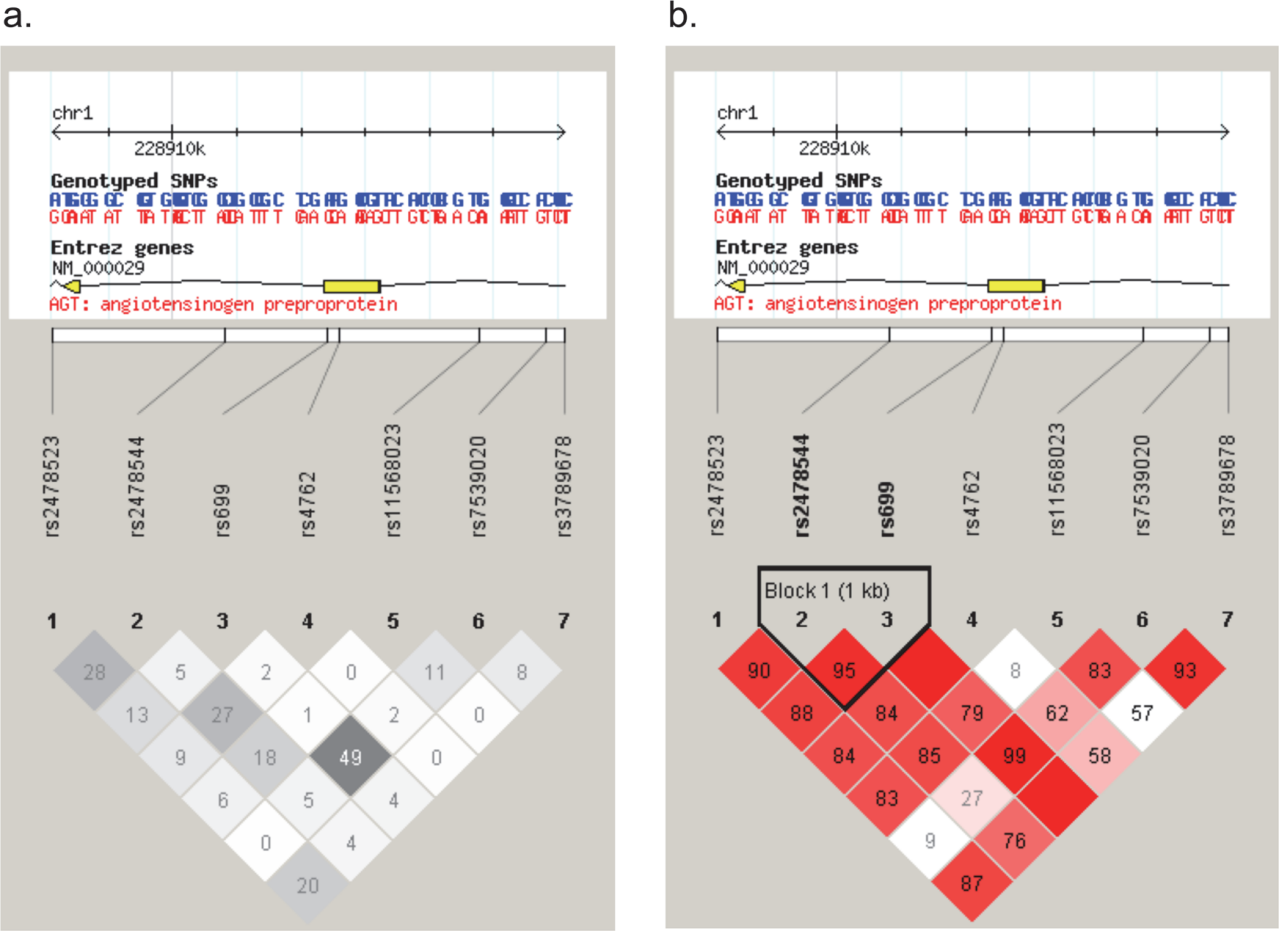
SNP locations and LD structure of AGTgene between AF group and no-AF heart disease control group. Each figure was composed of chromosome scale (the top line with even division), the transcription string (the thick bars represent exon (yellow) or UTR (blue), the thin lines represent intron), SNP scale (the hollow bar with scales representing SNPs location), and graphic of LD (black-and-white) or block definition (flammulated).The measure of LD (r^2^) among all possible pairs of SNPs is shown graphically according to the shade of color (a),where white represents very low r^2^ and scarlet represents very high r^2^. The numbers in squares are r^2^values (r^2^ x 100).The measure of LD (D') among all possible pairs of SNPs is shown graphically according to the shade of color (b), where white represents very low D' and dark represents very high’. The numbers in squares are D' values (D' x 100).

**Fig 2:**
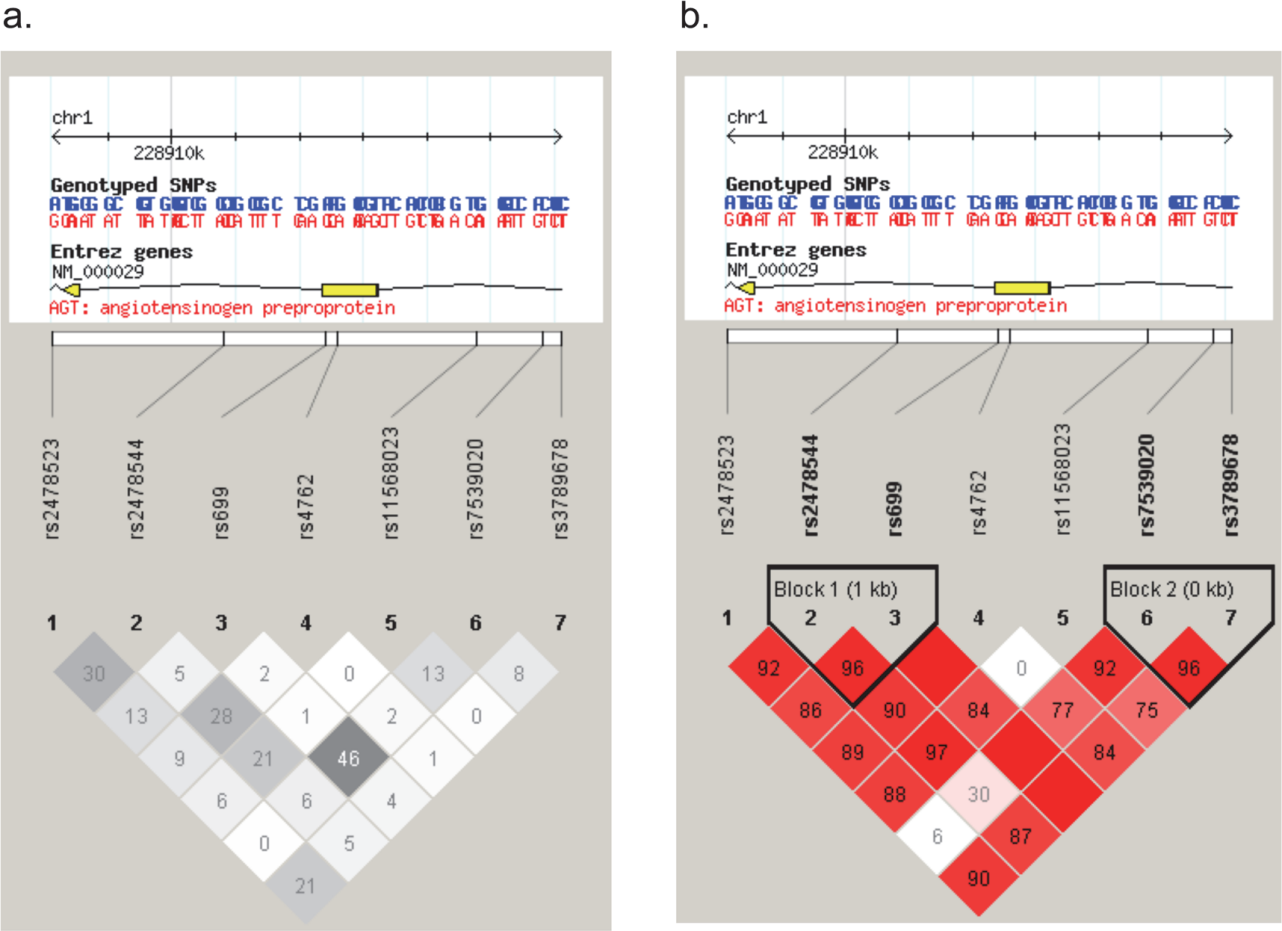
SNP locations and LD structure of AGTgene between AF group and healthy control group. Each figure was composed of chromosome scale (the top line with even division), the transcription string (the thick bars represent exon (yellow) or UTR (blue), the thin lines represent intron), SNP scale (the hollow bar with scales representing SNPs location), and graphic of LD (black-and-white) or block definition (flammulated).The measure of LD (r^2^) among all possible pairs of SNPs is shown graphically according to the shade of color (a), where white represents very low r^2^ and scarlet represents very high r^2^. The numbers in squares are r^2^values (r^2^ x 100).The measure of LD (D') among all possible pairs of SNPs is shown graphically according to the shade of color (b), where white represents very low D' and dark represents very high’. The numbers in squares are D' values (D' x 100).

The plots of the pairwise LD (D’) values for the tSNPs and LD structures of each gene are displayed in Figs. 1B, 2B. The LD plot indicates that compared with the non-AF heart disease control and the healthy control group, for AGT gene, block 1 (SNPs 2–3: size 1598bp) with high LD were identified, encompassing the downstream intron 1 and the former exon 2 (Figs. 1B, 2B); And compared with the healthy control group, block 2 (SNPs 6–7: size 292bp) with high LD were identified, encompassing the intron region (Fig. 2B). For CYP11B2 gene, compared with the healthy control group, we identified the following regions of strong LD: SNPs 1–2 (size 451bp) covering the 3’ UTR. The calculation of pairwise LD (r^2^) values for these tSNPs is illustrated in Figs. 1A, 2A.

### Haplotype Analysis

The associations between frequencies of the haplotypes and AF risk are showed in Table 6 and S1 Table. In AGT block 1, ‘CC’ haplotype (P = 0.048, OR = 0.819 and 95% CI = 0.672–0.998) and ‘GT’ haplotype (P = 0.015, OR = 0.747 and 95% CI = 0.590–0.944) were revealed asprotective role. And in AGT block 2, ‘TT’ haplotype (carrying rs3789678-T-allele) was associated with risk of AF, compared with the common ‘TC’ haplotype between the AF group and the healthy control group, (P = 0.013, OR = 1.332 and 95% CI = 1.063–1.669), adjusted for age, gender, LVEF, LVEDD, LAD and frequency of hypertension and diabetes (Table 6).

**Table 6:** Associations of common haplotypes of AGT gene and CYP11B2 gene with AF risk between AF group and healthy control group.

Block	Gene	Haplotype	Case	Frequency	Control	Frequency	P^a^	Hap. Score^b^	P_sim_ ^c^	P^d^	OR (95% CI)	Global score test
Block 1	AGT	rs2478544-rs699										
		GC	1191	63.96%	876	60.25%	0.223	1.21769	0.213		1.000 (referent)	Global-stat = 2.20621, df = 3, P = 0.53073, P_sim_ ^c^ = 0.50123
		CC	407	21.86%	345	23.73%	0.264	-1.11691	0.254	0.048	0.819 (0.672–0.998)	
		GT	262	14.07%	233	16.02%	0.755	-0.31144	0.750	0.015	0.747 (0.590–0.944)	
		CT	2	0.11%	0	0.00%	NA^e^	NA^e^	NA^e^	NA^e^	NA^e^	
Block 2	AGT	rs7539020-rs3789678										
		TC	944	50.70%	770	52.96%	0.4559	-0.74563	0.441		1.000 (referent)	Global-stat = 9.37498, df = 3, P = 0.0247, P_sim_ ^c^ = **0.01923**
		CC	561	30.13%	452	31.09%	0.1355	-1.49278	0.126	0.732	1.033 (0.858–1.243)	
		TT	354	19.01%	232	15.96%	0.0061	2.74155	0.005	**0.013**	**1.332 (1.063–1.669)**	
		CT	3	0.16%	0	0.00%	NA^e^	NA^e^	NA^e^	NA^e^	NA^e^	
Block 1	CYP11B2	rs3802230-rs3097										
		CG	1235	66.33%	944	64.92%	0.1462	1.45307	0.138		1.000 (referent)	Global-stat = 5.45791, df = 2, P = 0.06529, P_sim_ ^c^ = 0.05657
		AG	495	26.58%	426	29.30%	0.0283	-2.19335	0.025	0.059	0.837 (0.696–1.006)	
		CA	132	7.09%	84	5.78%	0.2298	1.20086	0.217	0.499	1.122 (0.801–1.569)	

a. P value for difference in haplotype frequency between AF group and healthy group.

b. A positive (or negative) score for a particular haplotype would have suggested that the haplotype was associated with increased (or decreased) AF risk

c. Generated by permutation test with 100,000 times simulation.

d. P values from unconditional logistic regression analyses, adjusted for age, gender, LVEF, LVEDD, LAD and frequency of hypertension and diabetes.

e. NA, not available because of the rarity of haplotype.

The significant associations of diplotypes in some blocks are listed in Table 7 and S2 Table. In AGT block 1, the diplotype with ‘GC’ was found AF risk (for two-copy, P = 0.036, OR = 1.450, 95% CI = 1.025–2.050); and the diplotype with ‘GT’ showed protective effects (for one-copy, P = 0.007, OR = 0.692, 95% CI = 0.528–0.906). In AGT block 2, the diplotype with ‘TC’, carrying rs3789678-C-allele, was associated with reduced risk of AF between the AF group and the healthy control group (for one-copy, P = 0.017, OR = 0.704, 95% CI = 0.528–0.939). The diplotype with ‘TT’ haplotype in the same block, carrying rs3789678-T-allele, was associated with increased risk of AF (for two-copy, P = 0.043, OR = 2.162 and 95% CI = 1.025–4.563). In CYP11B2 block, the diplotype with ‘AG’, carrying rs3097-G-allele was estimated as decreased risk of AF between the AF group and the healthy control group (for one-copy, P = 0.009, OR = 0.721, 95% CI = 0.564–0.921) (Table 7). These diplotype analyses were all adjusted for age, gender, LVEF, LVEDD, LAD and frequency of hypertension and diabetes.

**Table 7:** Diplotype analysis of AGT gene and CYP11B2 gene polymorphisms with AF risk between AF group and healthy control group.

Gene	Haplotype	0-copy		1-copy Logistic Regression			2-copy Logistic Regression			P (2 df)^b^	P_trend_
		case/control	OR (95%CI)	case/control	P^a^	OR (95%CI)	case/control	P^a^	OR (95%CI)		
AGT	rs2478544-rs699										
	GC	143/112	1.000 (referent)	385/354	0.990	1.002 (0.714–1.406)	403/261	**0.036**	**1.450 (1.025–2.050)**	0.0053	0.0326
	CC	586/426	1.000 (referent)	283/257	0.115	0.819 (0.638–1.050)	62/44	0.522	0.857 (0.533–1.376)	0.1016	0.2179
	GT	699/514	1.000 (referent)	202/193	**0.007**	**0.692 (0.528–0.906)**	30/20	0.882	1.056 (0.514–2.169)	0.0671	0.1286
	CT	929/663	1.000 (referent)	2/0	NA^c^	NA^c^	0/0	NA^c^	NA^c^	NA^c^	NA^c^
AGT	rs7539020-rs3789678										
	TC	239/156	1.000 (referent)	440/372	**0.017**	**0.704 (0.528–0.939)**	252/199	0.114	0.770 (0.557–1.064)	0.1148	0.2009
	CC	460/341	1.000 (referent)	381/320	0.424	0.906 (0.710–1.154)	90/66	0.924	0.980 (0.651–1.477)	0.4485	0.5533
	TT	608/506	1.000 (referent)	292/210	0.076	1.259 (0.976–1.624)	31/11	**0.043**	**2.162 (1.025–4.563)**	**0.0264**	**0.0195**
	CT	0/0	1.000 (referent)	2/0	NA^c^	NA^c^	0/0	NA^c^	NA^c^	NA^c^	NA^c^
CYP11B2	rs3802230-rs3097										
	CG	119/92	1.000 (referent)	389/326	0.318	0.829 (0.573–1.198)	423/309	0.606	1.102 (0.762–1.594)	0.4305	0.4087
	AG	513/368	1.000 (referent)	341/292	**0.009**	**0.721 (0.564–0.921)**	77/67	0.461	0.853 (0.559–1.302)	0.000016	0.0909
	CA	806/647	1.000 (referent)	118/76	0.587	1.104 (0.773–1.577)	7/4	0.247	2.469 (0.535–11.399)	0.3256	0.1363

a. P values from unconditional logistic regression analyses, adjusted for age, gender, LVEF, LAD, LVEDD and frequency of hypertension and diabetes.

b. Global P values [2 degrees of freedom (df)]: diplotype frequencies in AF cases and healthycontrols were compared using a χ^2^ test with 2 df.

c. NA, not available because of the rarity of haplotype.

## Discussion

Previous studies only focused on a few variants in RAS system genes, which may be insufficient to capture the full effects of susceptibility genes. This study targeted these main genes of RAS system (ACE, AGT, and CYP11B2 genes) and tested the hypothesis that genetic predisposition may be underlying the prevalence of nonfamilial AF by observing the association of tSNPs in RAS system gene with AF in a large pair-matched case control study.

Some studies reported that the polymorphisms of rs699 (M235T), rs5050 (A-20C), rs11568020 (G-152A), rs5051 (G-6A), rs4762 (T174M) and rs5049 (G-217A) were related to AF [2, 3, 10]. In the study, we found rs3789678 and rs11568023 of AGT gene exhibited statistically significant association with AF in Chinese Han population. Both of two sites had not been previously reported by other AF research groups. Rs11568023 and rs3789678 are in intron of the AGT gene. They showed no linkage disequilibrium (r^2^ < 0.8) with the published six variants in Han Chinese population. However, rs3789678 was in strong LD (r^2^ > 0.8) with four promoter SNPs (rs2071404, rs5046, rs11122580, rs2071405). Rs11568023 is localized in intronic region of AGT gene. It may be strongly linked with a potential functional locus or exerts molecular influence with AGT gene transcription. However, in HapMap database, the Han Chinese Beijing (HCB) sample size (about 90 healthy persons) is too small to show the virtual linked polymorphism of rs11568023. In addition, rs11568023 was only 1303bp away from rs3789678, but the function of these two polymorphisms was inverse. Consistently, in the haplotype and diplotype analysis “T” mutation of AGT gene rs3789678 was found associated with AF risk. Therefore, we demonstrated that the rs3789678 T allele in intron of the AGT gene and the corresponding haplotypes were associated with AF. A specific haplotype may be associated with higher AGT gene transcription activity. This higher transcription may cause a higher tissue angiotensin II concentration in the atrium, which subsequently causes atrial fibrosis, conduction heterogeneity and decreased atrial effective refractory period and provides the substrates for the development of AF.

In previous studies, rs699 proved to be associated with atrial fibrillation in Chinese Taiwan and Turkish population[2, 11], but it did not exhibit association in our sample set. However, it showed association as haplotype form. There are some reasons as follows: Chinese Taiwan population is the Gaoshan nationality, whereas the population living in Shanghai belongs to Han nationality. In addition to territory and population stratification, another possible reason is that sample size is still not big enough. Although the sample size of our study was larger than the studies in Chinese Taiwan and Turkish population, we still need to verify the result in a more large-scale population. Other studies revealed that-344C/T (rs1799998) of CYP11B2 was associated with AF risk [12, 13, 14]. In our study, the single “A” mutation of CYP11B2 rs3802230 in the diplotype analysis, which was no genetic linkage with reported polymorphisms, showed reduced risk of AF. The main physiological determinants of serum aldosterone concentration are volume status, potassium levels, and activity of the renin-angiotensin-aldosterone system [15]. Genetic variation, such as rs3802230 of CYP11B2 gene, might modulate the expression of the enzyme [16], and therefore contribute to the inherited variability of aldosterone levels.

Control subjects in our study consisted of both healthy individuals and patients with heart diseases. The only difference between the AF group and the control group consisting of patients with heart diseases was the presence or absence of AF. Although the groups were similar in respect of age, hypertension, diabetes, and heart disease. AF patients had a significantly larger left atrium, higher LVEDD, and low LVEF. These findings may imply that stretching of the atria is responsible for the development of AF. In the Framingham study, it was found that left atrial dilation and reduced left ventricular fractional shortening increased the risk of AF [17]. Because gender, left atrial size, LVEDD, and LVEF were not balanced in the AF group and the two control groups, we reanalyzed the associations between AF and RAS polymorphisms with adjustmentfor these parameters. Hypertension and diabetes are known phenotypes associated with polymorphisms in RAS genes [18]. In our study, the case and the healthy group were not matched with regard to these frequencies of hypertension and diabetes. Thus, we analyzedthe associations between AF and RAS polymorphisms between the case and the healthy group, adjusted for the frequency of hypertension and diabetes.

Some limitations are inherent in this study and must be noted. Although we used a statistical correction to adjust for multiple testing for a given gene, current epidemiologic and statistical literature is not unanimously clear on when and how to make such corrections. Although frequently used, both Bonferroni correction and Bayesian techniques [19] are problematic in correcting multiple comparisons. However, some authors believe that corrections were not needed when different associations in a study are of interest on a purely one-at-a-time basis [20, 21]. In addition, although our study suggested that RAS system gene was involved in the prevalence of nonfamilial AF, the tSNPs selected for this study were not sufficient in capturing all the geneticvariation in Chinese Hans. Therefore, we were not able to exclude the potential association between rare alleles and nonfamilial AF in Chinese Hans.

## Conclusion

Activation of the RAS may be an important risk factor for the occurrence and development of AF. In our study, we found that new polymorphisms/haplotypes in AGT gene were associated with nonfamilial AF in Chinese Han population. Therefore, genetic variation in the RAS system may influence effect of renin-angiotensin system blockers on atrial fibrillation. More importantly, this study shows the complexities of a multifactorial and multigenic disease like AF, and the necessity to continue studying it with a more comprehensive approach.

## Supporting Information

S1 Table Associations of common haplotypes of AGT gene with AF risk between AF group and non-AF heartdisease control group. (DOCX)

S2 Table Diplotype analysis of AGT gene polymorphisms with AF risk between AF group and non-AF heart disease control group. (DOCX)
