# Correction: Polymorphisms of Renin-Angiotensin-Aldosterone System Gene in Chinese Han Patients with Nonfamilial Atrial Fibrillation

**DOI:** 10.1371/journal.pone.0123847

**Published:** 2015-03-30

**Authors:** The PLOS ONE Staff

The seventh author’s name is spelled incorrectly. The correct name is Zhimin Wang.
