# Supplementary material for: Polymorphisms of Renin-Angiotensin-Aldosterone System Gene in Chinese Han Patients with Nonfamilial Atrial Fibrillation
Source: PLoS One. 2015 Feb 27;10(2):e0117489. doi: 10.1371/journal.pone.0117489 (PMC4344326; doi:10.1371/journal.pone.0117489)
Supplement: S1 Table — (DOCX) [file pone.0117489.s001.docx]

S1 Table. Associations of common haplotypes of AGT gene with AF risk between AF group and non-AF heartdisease control group.

| Block | Gene | Haplotype | Case | Frequency | Control | Frequency | P^a^ | Hap. Score^b^ | P_sim_^c^ | P^d^ | OR (95% CI) | Global score test |
| --- | --- | --- | --- | --- | --- | --- | --- | --- | --- | --- | --- | --- |
| Block 1 | AGT | rs2478544-rs699 | | | | | | | | | | |
|  |  | GC | 1194 | 64.12% | 875 | 65.20% | 0.443 | 0.76643 | 0.445 |  | 1.000 (referent) | Global-stat = 1.29876, df = 3, P = 0.72943, P_sim_^c^ =0.75969 |
|  |  | CC | 407 | 21.86% | 279 | 20.79% | 0.842 | -0.20003 | 0.839 | 0.847 | 1.018 (0.848-1.222) |  |
|  |  | GT | 259 | 13.91% | 188 | 14.01% | 0.414 | -0.81765 | 0.412 | 0.887 | 0.984 (0.793-1.222) |  |
|  |  | CT | 2 | 0.11% | 0 | 0.00% | NA^e^ | NA^e^ | NA^e^ | NA^e^ | NA^e^ |  |

a. P value for difference in haplotype frequency between AF and non-AF heart disease group.

b. A positive (or negative) score for a particular haplotype would have suggested that the haplotype was associated with increased (or decreased) AF risk

c. Generated by permutation test with 100,000 times simulation.

d. P values from unconditional logistic regression analyses, adjusted for age, gender, LVEF, LAD and LVEDD.

e. NA, not available because of the rarity of haplotype.
