# Supplementary material for: Polymorphisms of Renin-Angiotensin-Aldosterone System Gene in Chinese Han Patients with Nonfamilial Atrial Fibrillation
Source: PLoS One. 2015 Feb 27;10(2):e0117489. doi: 10.1371/journal.pone.0117489 (PMC4344326; doi:10.1371/journal.pone.0117489)
Supplement: S2 Table — (DOCX) [file pone.0117489.s002.docx]

S2 Table. Diplotype analysis of AGT gene polymorphisms with AF risk between AF group and non-AF heart disea**se** control group.

| Gene | Haplotype | 0-copy | | 1-copy Logistic Regression | | | 2-copy Logistic Regression | | | P (2 df)^b^ | P_trend_ |
| --- | --- | --- | --- | --- | --- | --- | --- | --- | --- | --- | --- |
|  |  | case/control | OR (95%CI) | case/control | P^a^ | OR (95%CI) | case/control | P^a^ | OR (95%CI) |  |  |
| AGT | rs2478544-rs699 | | | | | | | | | | |
|  | GC | 142/106 | 1.000 (referent) | 384/255 | 0.220 | 1.214 (0.891-1.655) | 405/310 | 0.696 | 1.062 (0.784-1.440) | 0.4192 | 0.5541 |
|  | CC | 586/430 | 1.000 (referent) | 283/203 | 0.882 | 0.983 (0.782-1.235) | 62/38 | 0.661 | 1.103 (0.713-1.706) | 0.7037 | 0.4879 |
|  | GT | 702/502 | 1.000 (referent) | 199/150 | 0.635 | 0.941 (0.732-1.210) | 30/19 | 0.776 | 1.094 (0.589-2.031) | 0.8251 | 0.9391 |
|  | CT | 0/0 | 1.000 (referent) | 2/0 | NA^c^ | NA^c^ | 0/0 | NA^c^ | NA^c^ | NA^c^ | NA^c^ |

a. P values from unconditional logistic regression analyses, adjusted for age, gender, LVEF, LAD，LVEDD.

b. Global P values [2 degrees of freedom (df)]: diplotype frequencies in AF group and non-AF heart disease control group were compared using a χ^2^ test with 2 df.

c. NA, not available because of the rarity of haplotype
